# Supplementary material for: Causal Relationship between Aging and Anorexia Nervosa: A White-Matter-Microstructure-Mediated Mendelian Randomization Analysis
Source: Biomedicines. 2024 Aug 16;12(8):1874. doi: 10.3390/biomedicines12081874 (PMC11351342; doi:10.3390/biomedicines12081874)
Supplement: Supplementary file 1 [file biomedicines-12-01874-s001.zip › Supplementary Figure/Figure S4/Singe SNP plot of MR analysis for TL to ubm-b-2062.pdf]

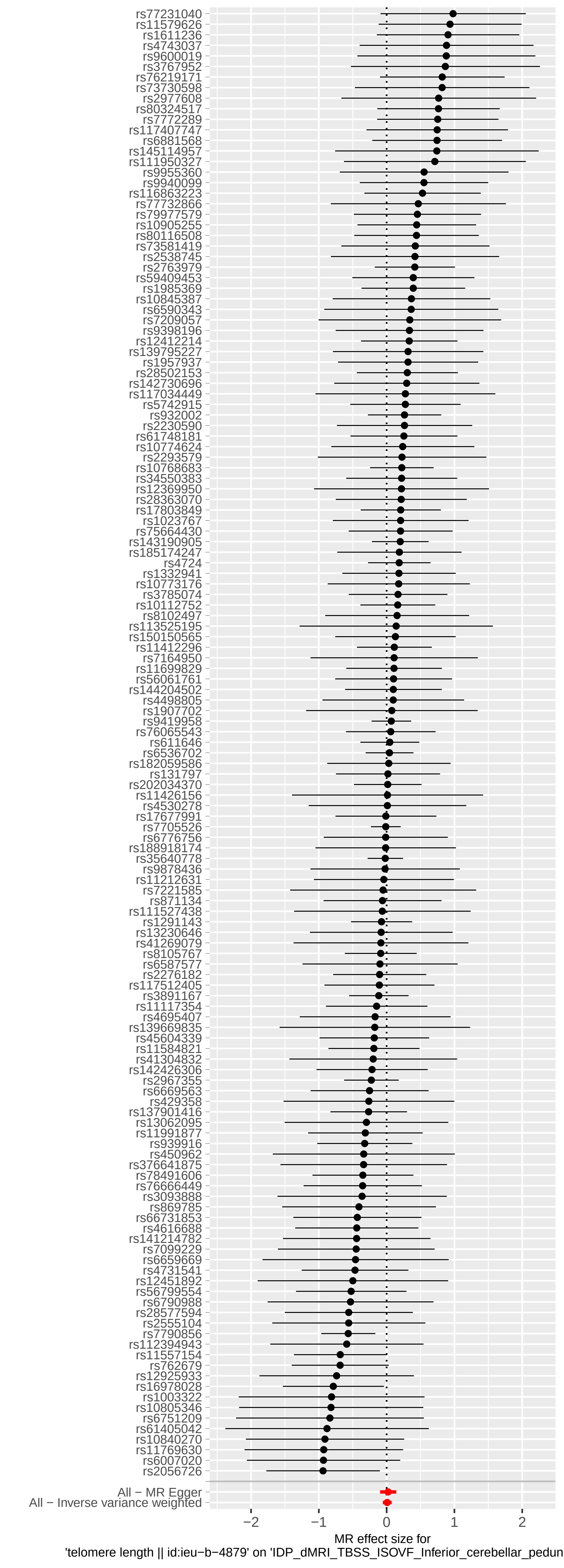
rs77231040
rs11579626
rs1611236
rs4743037
rs9600019
rs3767952
rs76219171
rs73730598
rs2977608
rs80324517
rs7772289
rs117407747
rs6881568
rs145114957
rs111950327
rs9955360
rs9940099
rs116863223
rs77732866
rs79977579
rs10905255
rs80116508
rs73581419
rs2538745
rs2763979
rs59409453
rs1985369
rs10845387
rs6590343
rs7209057
rs9398196
rs12412214
rs139795227
rs1957937
rs28502153
rs142730696
rs117034449
rs5742915
rs932002
rs2230590
rs61748181
rs10774624
rs2293579
rs10768683
rs34550383
rs12369950
rs28363070
rs17803849
rs1023767
rs75664430
rs143190905
rs185174247
rs4724
rs1332941
rs10773176
rs3785074
rs10112752
rs8102497
rs113525195
rs150150565
rs11412296
rs7164950
rs11699829
rs56061761
rs144204502
rs4498805
rs1907702
rs9419958
rs76065543
rs611646
rs6536702
rs182059586
rs131797
rs202034370
rs11426156
rs4530278
rs17677991
rs7705526
rs6776756
rs188918174
rs35640778
rs9878436
rs11212631
rs7221585
rs871134
rs111527438
rs1291143
rs13230646
rs41269079
rs8105767
rs6587577
rs2276182
rs117512405
rs3891167
rs11117354
rs4695407
rs139669835
rs45604339
rs11584821
rs41304832
rs142426306
rs2967355
rs6669563
rs429358
rs137901416
rs13062095
rs11991877
rs939916
rs450962
rs376641875
rs78491606
rs76666449
rs3093888
rs869785
rs66731853
rs4616688
rs141214782
rs7099229
rs6659669
rs4731541
rs12451892
rs56799554
rs6790988
rs28577594
rs2555104
rs7790856
rs112394943
rs11557154
rs762679
rs12925933
rs16978028
rs1003322
rs10805346
rs6751209
rs61405042
rs10840270
rs11769630
rs6007020
rs2056726
All − MR Egger
All − Inverse variance weighted
−2
−1
0
1
2
MR effect size for
'telomere length || id:ieu−b−4879' on 'IDP_dMRI_TBSS_ISOVF_Inferior_cerebellar_pedun
